# Machine learning models for predicting liver cancer: a real-world cohort study in China

**DOI:** 10.3389/fcell.2026.1896989

**Published:** 2026-09-09

**Authors:** Cexiong Fu, Fang Li, Shibing Li, Ruifeng Lin, Zhao Yuan

**Affiliations:** 1 Department of Hepatobiliary Pancreatic Surgery, Hainan General Hospital, Hainan Affiliated Hospital of Hainan Medical University, Haikou, China; 2 Department of Gastroenterology, Gastroenterology Endoscopy Center, Hainan General Hospital, Hainan Affiliated Hospital of Hainan Medical University, Haikou, China; 3 Department of Pediatric Surgery, Hainan General Hospital, Hainan Affiliated Hospital of Hainan Medical University, Haikou, China; 4 Department of Traditional Chinese Internal Medicine, School of Traditional Chinese Medicine, Southern Medical University, Guangzhou, China; 5 The Second Affiliated Hospital of Guilin Medical University, Guilin, China

**Keywords:** auxiliary diagnosis, liver cancer, machine learning, predictive model, XGBoost

## Abstract

**Background:**

Liver cancer has high incidence and mortality worldwide, and timely identification is important for improving prognosis. However, prediction models based on routinely available clinical data remain insufficiently evaluated in hospitalized real-world populations. This study aimed to develop and interpret a machine-learning model for liver cancer prediction using multidimensional clinical and laboratory features.

**Methods:**

This retrospective real-world cohort included 9,284 hospitalized patients from Hainan General Hospital between January 2020 and December 2023. Patients were divided into a training set (n = 6,426) and an internal validation set (n = 2,858). Seventy-four demographic, clinical, and laboratory variables were collected. Feature selection was performed using least absolute shrinkage and selection operator regression in the training set. Six models were developed: logistic regression, random forest, support vector machine, k-nearest neighbors, extreme gradient boosting (XGBoost), and Elastic Net. Discrimination was assessed primarily using the area under the receiver operating characteristic curve (AUC). Decision curve analysis evaluated clinical net benefit, and SHapley Additive exPlanations (SHAP) were used for model interpretation.

**Results:**

Liver cancer accounted for 48.7% of the training cohort and 48.8% of the validation cohort. LASSO retained 57 nonzero model terms at the minimum-error penalty. XGBoost achieved the highest AUC among all models, with an AUC of 0.909 (95% CI, 0.903–0.915) in the training set and 0.802 (95% CI, 0.788–0.817) in the validation set. At a threshold of 0.5, validation accuracy, sensitivity, specificity, and F1 score were 0.730, 0.758, 0.698, and 0.753, respectively. Decision curve analysis showed that XGBoost provided the greatest net clinical benefit across a broad range of threshold probabilities. SHAP analysis identified serum sialic acid, the aspartate aminotransferase-to-alanine aminotransferase ratio, alkaline phosphatase, basophil percentage, and monocyte-to-lymphocyte ratio as the leading predictors.

**Conclusion:**

XGBoost showed good discrimination and interpretability for liver cancer prediction in a hospitalized real-world cohort using routinely available clinical and laboratory data. The findings support the feasibility of leveraging large-scale inpatient laboratory data for risk-stratification model development. Serum sialic acid, the aspartate aminotransferase-to-alanine aminotransferase ratio, and alkaline phosphatase were important predictors. Prospective validation in outpatient, first-visit, high-risk, and external populations is required before broader clinical or screening application.

## 1 Introduction

Liver cancer is one of the most common malignancies worldwide and imposes a substantial public health burden because of its high incidence and mortality. According to GLOBOCAN 2020 data, liver cancer was among the top three causes of cancer-related death in 46 countries and among the top five causes in 90 countries globally (Rumgay et al., 2022a; Bray et al., 2024). Hepatocellular carcinoma (HCC), the predominant subtype of primary liver cancer, accounts for approximately 80% of liver cancer cases worldwide and has a particularly high incidence in East Asia (Rumgay et al., 2022b). In China, approximately 367,000 new liver cancer cases and 316,000 deaths were projected in 2022. Liver cancer therefore remains a leading cause of cancer-related mortality and a persistent clinical burden (Chen et al., 2016; Han et al., 2024).

The high mortality rate of liver cancer is closely linked to insufficient early detection; most patients are diagnosed at intermediate or advanced stages, when the 5-year survival rate drops below 5%. In contrast, patients diagnosed with early-stage HCC who receive curative treatment can achieve a 5-year survival rate exceeding 70% (Parikh et al., 2023; Wang et al., 2023; Johnson et al., 2017). Improving early detection is therefore important for patient prognosis and overall survival (Johnson et al., 2017; Sohn et al., 2022). Current international guidelines, including those from the American Association for the Study of Liver Diseases and the European Association for the Study of the Liver, recommend abdominal ultrasound, with or without alpha-fetoprotein (AFP) testing, every 6 months for high-risk populations such as patients with cirrhosis or chronic hepatitis B infection (Heimbach et al., 2018; European Association for the Study of the Liver, 2018). Although this strategy has improved early detection, its sensitivity remains limited. Ultrasound has reduced sensitivity for early-stage HCC, particularly in obese patients and those with non-viral liver disease (Parikh et al., 2023; Singal et al., 2023). AFP also has suboptimal performance because it may remain normal in some patients with liver cancer and may be elevated in benign liver diseases (Wang et al., 2023; European Association for the Study of the Liver, 2018). These limitations highlight the need for complementary approaches to early liver cancer risk identification.

Recent advances in biomarker research and artificial intelligence (AI) have created new opportunities for liver cancer risk assessment. For example, the GALAD score, which combines sex, age, and three serum biomarkers, has shown good diagnostic performance for early HCC detection in multiple studies (Johnson et al., 2014; Best et al., 2020). Machine-learning and deep-learning approaches based on radiomics, digital pathology, and multimodal data have also shown promising performance in liver cancer screening, diagnosis, and prognosis prediction (Seif El Dahan et al., 2025; Wang et al., 2026; Zhang et al., 2024; Zhang et al., 2020). For example, the deep-learning model developed by Okanoue et al. achieved 85.3% sensitivity and 85.1% specificity for screening MAFLD-related HCC (Okanoue et al., 2023). However, current AI models for liver cancer screening still face important limitations, including limited generalizability across populations and reliance on imaging data, which may restrict accessibility in primary care settings (Chierici et al., 2025; Rao et al., 2026).

Existing liver cancer screening methods have important limitations, whereas emerging biomarkers and AI models may provide complementary tools. In this study, we integrated multidimensional clinical and laboratory indicators from hospitalized patients at a single tertiary hospital and used machine learning algorithms to construct an interpretable predictive model for liver cancer. We aimed to examine whether large-scale inpatient laboratory data could support low-cost liver cancer risk prediction while recognizing that validation in outpatient, first-visit, community-based, and external populations remains necessary.

## 2 Methods

### 2.1 Study design and population

This retrospective cohort study used data from hospitalized patients at Hainan General Hospital between January 2020 and December 2023. The Hainan General Ethics Committee reviewed and approved the study (Approval No. EC-YLY-2026-83-01). The study population comprised hospitalized patients who met the diagnostic criteria for liver cancer during the study period and contemporaneous hospitalized non-liver cancer controls. The control group included patients admitted for liver-related and non-liver-related conditions who had no liver cancer diagnosis in the electronic medical record. To improve the clinical interpretability of model specificity, we mapped the disease spectrum of non-liver cancer controls from electronic diagnosis codes and summarized it in Supplementary Table S2. This disease-spectrum analysis also clarified whether controls represented healthy individuals or non-liver cancer patients with liver disease or liver cancer-related risk backgrounds.

The diagnosis of liver cancer was required to conform to the relevant Chinese guidelines for the diagnosis and treatment of primary liver cancer (Zhou et al., 2020) and was confirmed by contrast-enhanced CT/MRI and/or pathological findings. In addition, the discharge diagnosis was required to include the corresponding code from the International Statistical Classification of Diseases and Related Health Problems, 10th Revision (ICD-10). The inclusion criteria were as follows: (1) age ≥18 years; (2) completion of relevant laboratory tests during hospitalization; and (3) complete clinical data. The exclusion criteria were as follows (Wang et al., 2025; Feng et al., 2026): (1) incomplete clinical or laboratory data; (2) other active malignancies; and (3) concomitant autoimmune diseases requiring long-term glucocorticoid or immunosuppressive therapy.

### 2.2 Data collection and outcome definition

Patient data were extracted from electronic medical records from the hospital. The outcome measure was a confirmed diagnosis of liver cancer. The collected data included demographic characteristics (age and sex), laboratory indicators (complete blood count, liver function, renal function, coagulation function, inflammatory markers, and tumor markers), and clinical features (liver cirrhosis, ascites, and hepatic encephalopathy). To ensure data quality, all variables were extracted based on the original records from the electronic medical system and independently cross-checked by two researchers.

### 2.3 Variables and data preprocessing

This study included 74 multidimensional features in three major categories: demographics, laboratory test results, and clinical characteristics. Outliers in continuous variables were identified using the 1.5 × interquartile range (IQR) rule. Specifically, values below Q1 − 1.5 × IQR or above Q3 + 1.5 × IQR were treated as outliers and set to missing before imputation. Features with a missing rate exceeding 40% were excluded from the analysis. For the remaining variables with missing data, continuous variables were imputed using multiple imputation by chained equations (MICE) with predictive mean matching (PMM), generating five imputed datasets with a maximum of 10 iterations. Missing categorical values were treated as a separate “missing” category where applicable. Missing-data handling was performed after dataset splitting and independently within the training and internal validation sets to reduce the risk of information leakage. Categorical variables were converted to factors, continuous variables were converted to numeric types, and variable names were standardized using the make.names function. Variables included in model development were required to have nonzero variance in the training set.

### 2.4 Model training and selection process

The dataset was split into training and internal validation sets at a 7:3 ratio using outcome-stratified random sampling with a fixed random seed. This split preserved similar liver cancer proportions in the two cohorts. Feature selection was performed exclusively in the training set using LASSO regression with 5-fold cross-validation (Xi et al., 2023). The minimum-error criterion (lambda.min; λ = 8.49 × 10^−4^), defined as the penalty parameter that minimized the cross-validated binomial deviance, was used rather than lambda.1se. Model terms with nonzero coefficients at lambda.min were retained for subsequent model construction. To evaluate collinearity, the correlation structure of continuous variables was examined using Spearman correlation (Supplementary Figure S1), and variance inflation factors (VIFs) were calculated for the retained terms. Feature-selection robustness was further assessed using bootstrap resampling of the LASSO procedure (Supplementary Figure S2).

**FIGURE 1:**
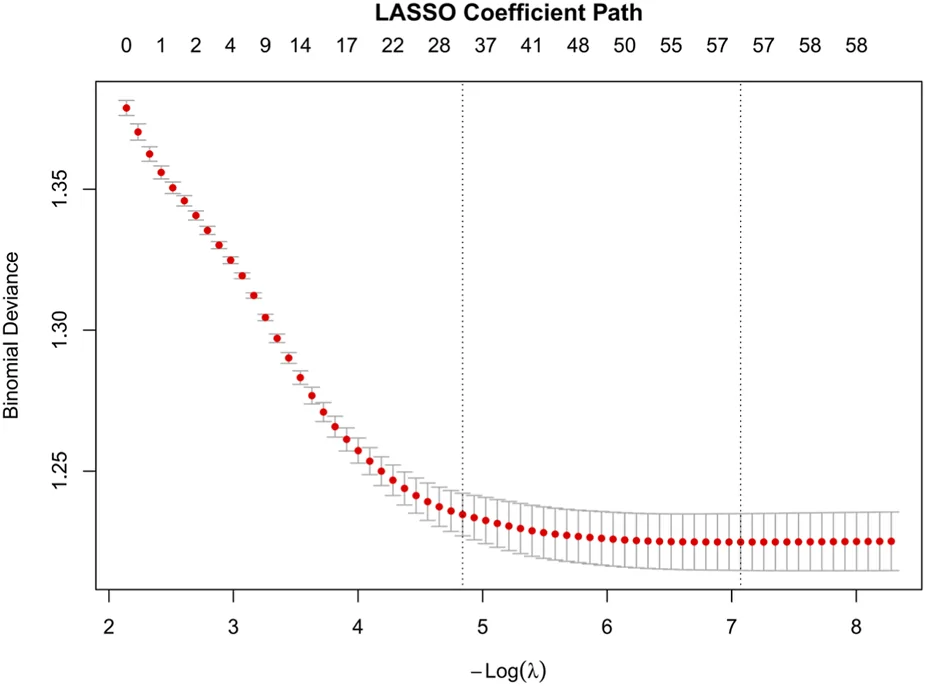
Feature selection using least absolute shrinkage and selection operator (LASSO) regression. The x-axis shows −log(λ), and the y-axis shows cross-validated binomial deviance. Numbers above the plot indicate the number of nonzero coefficients at each penalty value. The vertical reference lines indicate lambda.min and lambda.1se; the minimum-error criterion (lambda.min; λ = 8.49 × 10^−4^) was used for feature selection.

**FIGURE 2:**
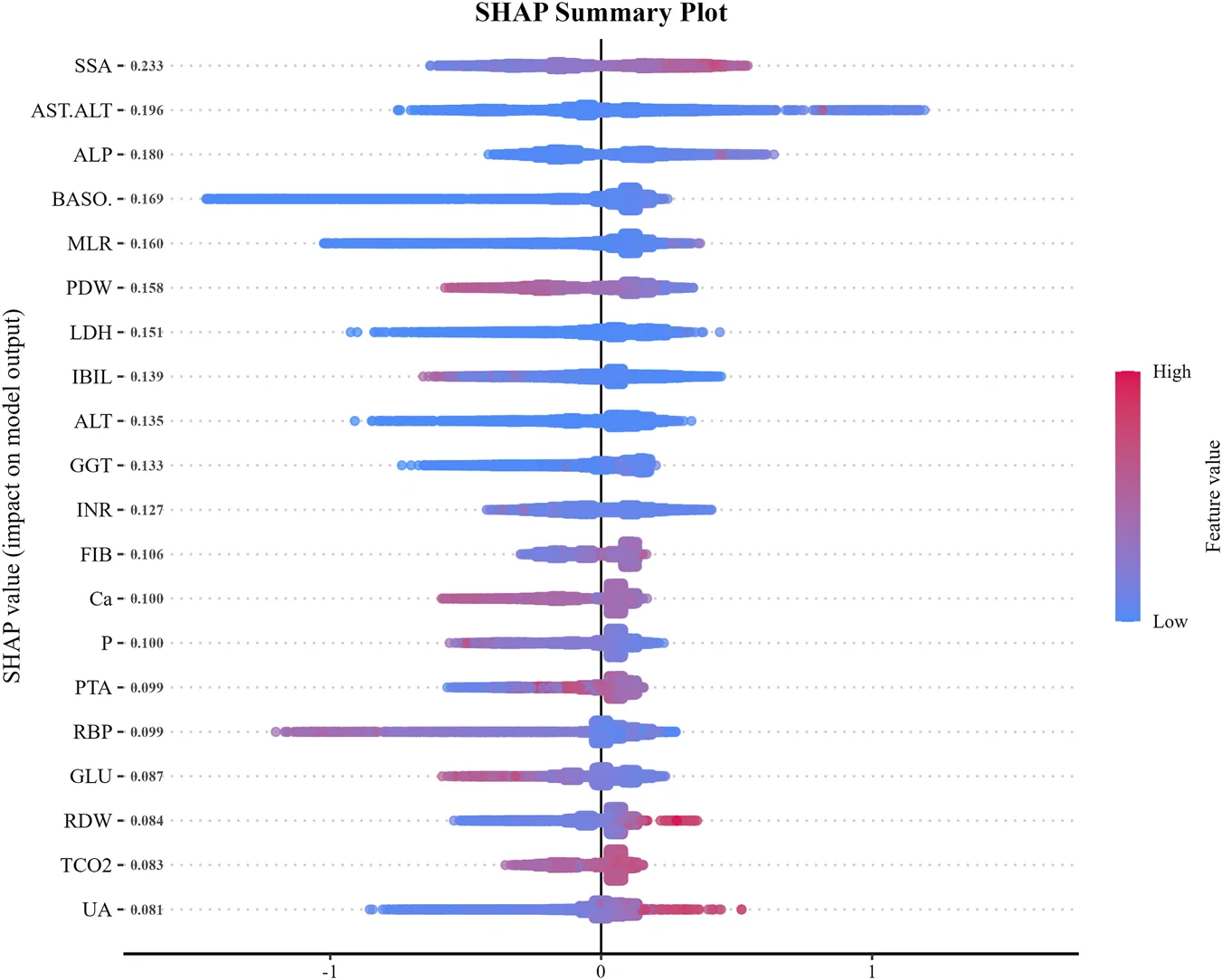
SHapley Additive exPlanations (SHAP) summary plot for the XGBoost model. Each point represents a patient-level SHAP value for one feature. The x-axis indicates the direction and magnitude of the feature contribution to the model output, and color represents the original feature value from low to high. Positive SHAP values indicate contributions toward a higher model-predicted probability of liver cancer.

**FIGURE 3:**
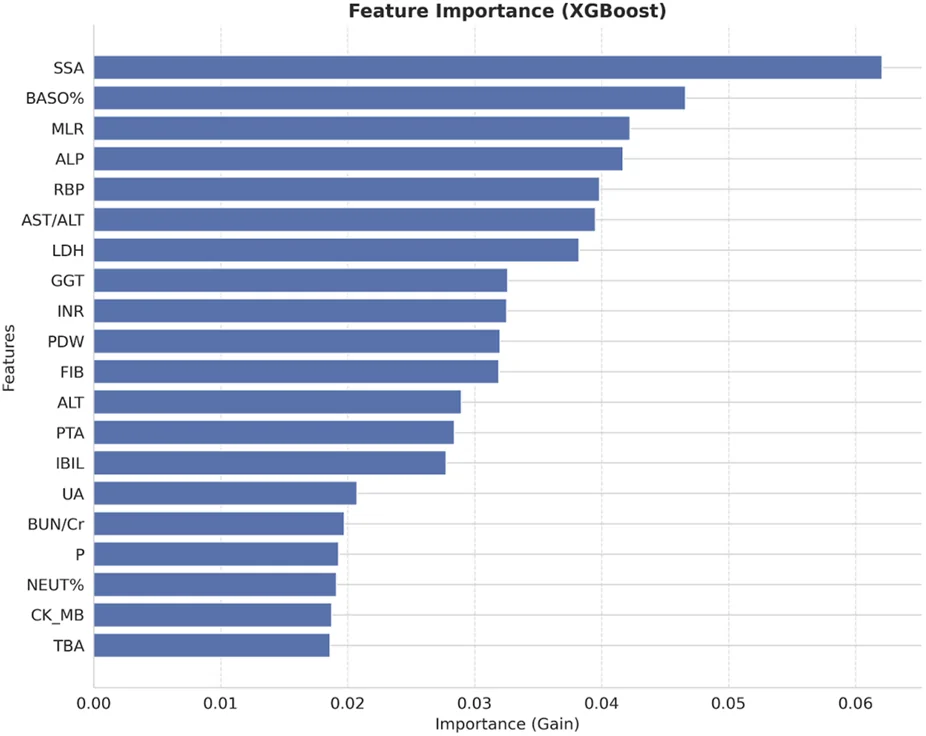
Gain-based feature-importance ranking for the XGBoost model. Features are ordered according to gain, which represents the average improvement in tree-split performance attributable to each feature. Larger gain values indicate greater contribution to model construction.

The retained feature list, LASSO coefficients, VIF diagnostics, bootstrap sensitivity results, correlation heatmap, LASSO stability plots, and model-importance rankings are reported in the Section 3, Supplementary Table S3, Supplementary Figures S1, S2, and Figures 1–3 to improve transparency and reproducibility.

Six classification models were developed using the retained terms: logistic regression, random forest, support vector machine (SVM), k-nearest neighbors (KNN), extreme gradient boosting (XGBoost), and Elastic Net. Model tuning was performed within the training set using 5-fold cross-validation where applicable. Logistic regression was fitted using a binomial family. Random forest was configured with 500 trees. SVM used a radial basis function kernel with probability estimates enabled. For KNN, the optimal k value was selected by cross-validation. XGBoost was trained using the binary:logistic objective and AUC as the evaluation metric, with max_depth = 3, eta = 0.05, subsample = 0.7, colsample_bytree = 0.7, and gamma = 0.1. Elastic Net was configured with alpha = 0.5, and the optimal lambda was selected by cross-validation. All feature-selection and model-tuning procedures were conducted within the training set; the internal validation set was reserved for model evaluation.

### 2.5 Comprehensive evaluation of models and model explanation

The primary model-performance metric was the area under the receiver operating characteristic (ROC) curve. The 95% confidence intervals (CIs) for AUCs were calculated using the DeLong method (Zou et al., 2024). Pairwise DeLong tests were performed in the internal validation cohort to compare XGBoost with each of the other five models, and the resulting P values were adjusted using the Benjamini-Hochberg procedure. Additional performance metrics, including accuracy, sensitivity, specificity, and F1 score, were calculated at a prespecified probability threshold of 0.5. Positive predictive value (PPV) and negative predictive value (NPV) were also evaluated for XGBoost under the prespecified threshold and exploratory alternative threshold strategies. Because threshold selection influences clinical utility, decision curve analysis (DCA) was used to evaluate net benefit across threshold probabilities (Netto Flores Cruz and Korthauer, 2024). All metrics were calculated separately for the training and validation sets. SHapley Additive exPlanations (SHAP) was applied to the optimal model for global interpretation. SHAP values quantify feature contributions to individual predictions and were interpreted as model-based associations rather than evidence of biological causality.

### 2.6 Statistical analysis

All statistical analyses were performed using R 4.0.1 (https://www.r-project.org). Continuous variables were summarized as medians with interquartile ranges (IQRs). Normality was assessed using the Shapiro-Wilk test. Normally distributed continuous variables were compared using Student’s t-test, whereas non-normally distributed continuous variables were compared using the Mann-Whitney U test. Categorical variables were expressed as frequencies and percentages and compared using the chi-square test or Fisher’s exact test, as appropriate. Statistical significance was defined as a two-tailed P < 0.05.

## 3 Results

### 3.1 Study population and baseline characteristics

A total of 9,284 hospitalized patients were enrolled in this study. The training set comprised 6,426 patients, including 3,130 (48.7%) with liver cancer, and the internal validation set comprised 2,858 patients, including 1,394 (48.8%) with liver cancer. This near-balanced case proportion facilitated model development and internal evaluation but differs from the prevalence expected in population-based screening settings. Diagnosis-code mapping showed that the non-liver-cancer control records were dominated by cirrhosis/chronic liver disease (2,167 records, 34.2%), viral hepatitis with cirrhosis (1,672 records, 26.4%), viral hepatitis without recorded cirrhosis (417 records, 6.6%), and endocrine, metabolic, or nutritional diseases (250 records, 3.9%) (Supplementary Table S2). Because this disease-spectrum summary was based on diagnosis records, the category counts should not be interpreted as unique-patient counts. Overall, the controls were not healthy population controls but hospitalized patients without liver cancer, many of whom had underlying liver disease or liver-cancer-related risk backgrounds.

Within the training set, patients in the liver cancer group had a median age of 57.0 years, which was significantly higher than that in the non-liver cancer group (55.0 years). The proportion of male patients was significantly higher in the liver cancer group than in the non-liver cancer group (P < 0.01). Regarding laboratory indicators, the liver cancer group exhibited significantly higher neutrophil counts, platelet counts, ALT, AST, GGT, ALP, and AFP and significantly lower lymphocyte percentage, albumin, albumin/globulin ratio, prothrombin time, and INR than the non-liver cancer group (all P < 0.01). The demographic and clinical characteristics of the patients with and without liver cancer were presented in Table 1.

**TABLE 1:** Baseline characteristics of the study population.

Variables	Internal training cohort (N = 6,426)		P value	Internal validation cohort (N = 2,858)		P value
	Non-liver cancer (n = 3296, 51.3%)	Liver cancer (n = 3130, 48.7%)		Non-liver cancer (n = 1464, 51.2%)	Liver cancer (n = 1394, 48.8%)	
Age, median (IQR), years	55.0 [45.0–62.0]	57.0 [49.0–65.0]	<0.01	55.0 [45.0–63.0]	57.0 [49.0–64.0]	<0.01
Male, n (%)	2685 (81.5%)	2829 (90.4%)	<0.01	1183 (80.8%)	1280 (91.8%)	<0.01
Ascites, n (%)	213 (6.5%)	231 (7.4%)	0.161	110 (7.5%)	114 (8.2%)	0.555
Upper Gastrointestinal Bleeding, n (%)	31 (0.9%)	10 (0.3%)	<0.01	12 (0.8%)	6 (0.4%)	0.281
Hepatic Encephalopathy, n (%)	111 (3.4%)	55 (1.8%)	<0.01	35 (2.4%)	34 (2.4%)	1
Liver Cirrhosis, n (%)	1924 (58.4%)	1797 (57.4%)	0.45	874 (59.7%)	844 (60.5%)	0.672
Liver Failure, n (%)	191 (5.8%)	88 (2.8%)	<0.01	88 (6.0%)	34 (2.4%)	<0.01
Neutrophil Percentage, median (IQR), %	59.30 [49.70–70.70]	63.40 [52.20–73.90]	<0.01	59.10 [49.10–70.90]	64.10 [53.10–75.00]	<0.01
Absolute Neutrophil Count, median (IQR), ×10^9^/L	2.87 [1.81–4.62]	3.37 [2.17–5.40]	<0.01	2.87 [1.81–4.72]	3.48 [2.22–5.41]	<0.01
Monocyte Percentage, median (IQR), %	9.60 [7.50–12.20]	10.10 [8.00–12.30]	<0.01	9.60 [7.50–12.25]	9.90 [8.00–12.20]	0.034
Absolute Monocyte Count, median (IQR), ×10^9^/L	0.48 [0.32–0.70]	0.55 [0.40–0.77]	<0.01	0.50 [0.34–0.72]	0.56 [0.39–0.78]	<0.01
Basophil Percentage, median (IQR), %	0.40 [0.20–0.70]	0.50 [0.30–0.70]	<0.01	0.40 [0.20–0.60]	0.50 [0.30–0.70]	<0.01
Absolute Basophil Count, median (IQR), ×10^9^/L	0.02 [0.01–0.03]	0.03 [0.02–0.04]	<0.01	0.02 [0.01–0.03]	0.03 [0.02–0.04]	<0.01
Eosinophil Percentage, median (IQR), %	2.20 [1.00–3.80]	2.20 [0.90–4.10]	0.708	2.10 [1.00–3.80]	2.25 [0.90–4.27]	0.329
Absolute Eosinophil Count, median (IQR), ×10^9^/L	0.10 [0.05–0.18]	0.11 [0.05–0.21]	<0.01	0.10 [0.05–0.19]	0.11 [0.05–0.21]	<0.01
Large Platelet Ratio, median (IQR)	0.33 [0.27–0.39]	0.31 [0.25–0.36]	<0.01	0.33 [0.27–0.40]	0.31 [0.26–0.36]	<0.01
Mean Corpuscular Volume, median (IQR), fL	91.70 [85.80–96.90]	92.30 [86.80–96.90]	0.071	91.30 [85.45–96.75]	91.50 [85.90–96.40]	0.963
Mean Corpuscular Hemoglobin Concentration, median (IQR), g/L	331.00 [321.00–340.00]	330.00 [321.00–338.75]	0.039	330.00 [320.00–340.00]	330.00 [321.00–338.00]	0.449
Mean Corpuscular Hemoglobin, median (IQR), pg	30.60 [28.10–32.50]	30.60 [28.30–32.40]	0.818	30.60 [27.90–32.30]	30.30 [28.20–32.20]	0.388
Lymphocyte Percentage, median (IQR), %	26.20 [16.55–35.10]	21.80 [13.60–31.67]	<0.01	26.60 [16.50–35.40]	20.90 [13.00–30.60]	<0.01
Absolute Lymphocyte Count, median (IQR), ×10^9^/L	1.20 [0.78–1.71]	1.15 [0.78–1.59]	<0.01	1.25 [0.78–1.71]	1.12 [0.80–1.53]	<0.01
White Blood Cell Count, median (IQR), ×10^9^/L	5.04 [3.55–7.06]	5.54 [4.07–7.73]	<0.01	5.12 [3.66–7.18]	5.64 [4.09–7.61]	<0.01
Red Blood Cell Count, median (IQR), ×10^12^/L	3.94 [3.13–4.64]	4.08 [3.46–4.65]	<0.01	3.96 [3.14–4.66]	4.08 [3.52–4.65]	<0.01
Red Cell Distribution Width, median (IQR), %	14.60 [13.20–17.20]	14.80 [13.40–17.00]	0.071	14.60 [13.30–17.30]	14.80 [13.40–17.10]	0.312
Hematocrit, median (IQR), L/L	0.36 [0.28–0.41]	0.37 [0.32–0.42]	<0.01	0.36 [0.29–0.42]	0.37 [0.32–0.42]	<0.01
Platelet Distribution Width, median (IQR), fL	13.50 [11.60–15.80]	12.50 [11.00–14.30]	<0.01	13.60 [11.70–16.10]	12.50 [11.10–14.40]	<0.01
Plateletcrit, median (IQR), %	0.13 [0.08–0.19]	0.15 [0.10–0.21]	<0.01	0.13 [0.08–0.19]	0.15 [0.10–0.21]	<0.01
Mean Platelet Volume, median (IQR), fL	11.00 [10.20–11.80]	10.70 [10.10–11.50]	<0.01	11.00 [10.20–11.90]	10.70 [10.10–11.40]	<0.01
Platelet Count, median (IQR), ×10^9^/L	108.00 [67.00–168.00]	134.00 [89.00–195.00]	<0.01	106.00 [68.00–167.00]	133.00 [89.00–193.00]	<0.01
Hemoglobin, median (IQR), g/L	118.00 [92.00–139.00]	123.00 [104.00–138.00]	<0.01	117.00 [93.00–139.00]	122.00 [105.00–137.00]	<0.01
Prealbumin, median (IQR), mg/L	120.00 [75.00–187.00]	115.00 [71.00–166.00]	<0.01	120.00 [74.00–185.25]	113.00 [68.50–167.00]	<0.01
Total Bile Acid, median (IQR), umol/L	18.00 [6.20–52.60]	16.90 [7.45–40.60]	0.206	16.75 [6.10–48.10]	17.40 [8.10–41.20]	0.247
Total Bilirubin, median (IQR), umol/L	19.11 [11.80–40.82]	17.66 [10.74–31.50]	<0.01	18.84 [11.69–39.05]	17.96 [10.65–30.54]	<0.01
Total Protein, median (IQR), g/L	65.70 [59.70–71.40]	65.90 [60.70–70.50]	0.366	66.00 [60.00–72.23]	65.70 [61.10–70.60]	0.91
Globulin, median (IQR), g/L	30.50 [26.60–34.90]	32.10 [28.10–37.20]	<0.01	30.90 [27.20–35.30]	32.20 [28.40–36.80]	<0.01
Albumin, median (IQR), g/L	33.90 [29.20–38.70]	33.10 [28.80–36.70]	<0.01	33.90 [29.10–39.30]	33.00 [28.78–36.90]	<0.01
Albumin/Globulin Ratio, median (IQR)	1.14 [0.89–1.38]	1.03 [0.82–1.25]	<0.01	1.12 [0.88–1.39]	1.02 [0.81–1.24]	<0.01
Direct Bilirubin, median (IQR), umol/L	7.36 [3.99–18.68]	7.20 [3.86–15.59]	<0.01	7.44 [3.88–17.74]	7.18 [4.05–14.77]	0.163
Alanine Aminotransferase, median (IQR), U/L	32.50 [21.50–57.20]	40.50 [25.70–70.75]	<0.01	32.90 [21.90–60.25]	40.30 [25.70–70.90]	<0.01
Aspartate Aminotransferase, median (IQR), U/L	42.00 [28.50–75.05]	58.70 [36.00–115.20]	<0.01	41.40 [28.20–75.25]	56.95 [36.50–112.40]	<0.01
Indirect Bilirubin, median (IQR), umol/L	11.46 [7.39–19.97]	10.11 [6.54–16.31]	<0.01	11.32 [7.31–19.45]	10.33 [6.55–16.32]	<0.01
Alpha-fetoprotein, median (IQR), ng/mL	3.93 [2.38–8.57]	23.47 [3.83–148.96]	<0.01	4.01 [2.57–9.70]	15.79 [3.57–148.66]	<0.01
Estimated Glomerular Filtration Rate, median (IQR), mL/min/1.73 m^2^	113.33 [91.93–135.86]	112.43 [93.20–134.57]	0.824	111.23 [89.60–132.43]	114.26 [94.34–137.45]	0.049
Urea, median (IQR), mmol/L	4.32 [3.35–5.65]	4.44 [3.53–5.65]	<0.01	4.41 [3.46–5.83]	4.42 [3.52–5.72]	0.942
Uric Acid, median (IQR), umol/L	292.00 [216.00–369.00]	299.00 [228.00–372.00]	<0.01	289.00 [211.00–367.75]	300.00 [224.25–372.00]	0.046
Total Carbon Dioxide, median (IQR), mmol/L	24.50 [22.80–26.25]	24.70 [23.10–26.30]	<0.01	24.80 [22.90–26.40]	24.60 [23.00–26.20]	0.666
Creatinine, median (IQR), umol/L	66.00 [55.00–79.00]	66.00 [56.00–77.00]	0.759	67.00 [56.00–79.25]	66.00 [56.00–78.00]	0.234
Retinol Binding Protein, median (IQR), mg/L	14.31 [8.32–22.88]	16.38 [10.25–23.79]	<0.01	14.05 [8.08–22.57]	15.78 [9.65–23.61]	<0.01
Alpha-Hydroxybutyrate Dehydrogenase, median (IQR), U/L	173.35 [144.17–218.43]	192.60 [157.70–248.80]	<0.01	172.50 [144.65–218.80]	190.10 [157.35–252.55]	<0.01
Lactate Dehydrogenase, median (IQR), U/L	219.40 [181.55–274.35]	243.95 [199.10–327.65]	<0.01	220.40 [183.60–285.10]	244.75 [198.25–318.62]	<0.01
Creatine Phosphokinase, median (IQR), U/L	89.40 [56.60–142.70]	82.60 [55.27–125.00]	<0.01	92.30 [57.90–148.25]	80.70 [52.48–128.50]	<0.01
Creatine Kinase-MB, median (IQR), U/L	26.50 [19.30–38.60]	27.60 [19.80–40.40]	<0.01	26.75 [18.90–40.98]	27.90 [20.00–41.20]	0.06
Glucose, median (IQR), mmol/L	5.20 [4.60–6.40]	4.90 [4.40–5.70]	<0.01	5.20 [4.60–6.30]	4.90 [4.40–5.70]	<0.01
C-Reactive Protein, median (IQR), mg/L	7.17 [2.65–25.88]	14.32 [4.19–46.47]	<0.01	8.06 [2.75–25.80]	13.89 [4.22–45.93]	<0.01
D-3-Hydroxybutyrate, median (IQR), mmol/L	0.11 [0.07–0.20]	0.12 [0.07–0.22]	0.032	0.11 [0.07–0.22]	0.12 [0.07–0.22]	0.344
Alpha-Fucosidase, median (IQR), U/L	30.30 [23.90–38.60]	35.10 [27.50–46.30]	<0.01	30.50 [23.50–39.20]	35.10 [27.40–45.80]	<0.01
Gamma-Glutamyl Transferase, median (IQR), U/L	50.60 [25.90–111.90]	90.10 [44.70–177.50]	<0.01	53.50 [26.63–115.87]	88.50 [44.05–163.00]	<0.01
Low-Density Lipoprotein Cholesterol, median (IQR), mmol/L	2.08 [1.53–2.74]	2.35 [1.78–3.05]	<0.01	2.09 [1.49–2.78]	2.33 [1.75–3.09]	<0.01
Homocysteine, median (IQR), umol/L	10.30 [8.00–12.80]	10.80 [8.60–13.30]	<0.01	10.40 [8.20–13.30]	10.80 [8.60–13.50]	0.038
Total Cholesterol, median (IQR), mmol/L	3.79 [2.91–4.63]	4.11 [3.27–4.97]	<0.01	3.74 [2.81–4.80]	4.10 [3.26–5.00]	<0.01
Chloride, median (IQR), mmol/L	106.00 [103.07–108.50]	105.00 [102.10–107.30]	<0.01	106.00 [102.80–108.50]	105.10 [101.50–107.47]	<0.01
Triglycerides, median (IQR), mmol/L	0.84 [0.63–1.17]	0.89 [0.66–1.22]	<0.01	0.86 [0.62–1.21]	0.85 [0.66–1.15]	0.972
Alkaline Phosphatase, median (IQR), U/L	89.80 [66.95–131.25]	113.60 [78.90–183.12]	<0.01	89.30 [67.00–129.20]	114.65 [79.62–180.93]	<0.01
Phosphorus, median (IQR), mmol/L	1.03 [0.88–1.18]	1.01 [0.88–1.15]	0.122	1.02 [0.88–1.17]	1.01 [0.87–1.15]	0.122
Cystatin C, median (IQR), mg/L	1.03 [0.86–1.23]	1.02 [0.87–1.23]	0.312	1.04 [0.88–1.25]	1.02 [0.86–1.21]	0.044
Calcium, median (IQR), mmol/L	2.13 [2.01–2.24]	2.15 [2.04–2.24]	<0.01	2.12 [2.01–2.22]	2.14 [2.04–2.24]	<0.01
Sodium, median (IQR), mmol/L	138.60 [136.10–140.50]	138.40 [135.80–140.40]	<0.01	138.50 [135.90–140.70]	138.40 [135.30–140.30]	0.049
Potassium, median (IQR), mmol/L	3.79 [3.52–4.06]	3.87 [3.61–4.15]	<0.01	3.78 [3.49–4.10]	3.88 [3.62–4.14]	<0.01
Magnesium, median (IQR), mmol/L	0.80 [0.74–0.86]	0.82 [0.76–0.87]	<0.01	0.80 [0.74–0.86]	0.82 [0.76–0.87]	<0.01
High-Density Lipoprotein Cholesterol, median (IQR), mmol/L	1.12 [0.84–1.40]	1.11 [0.84–1.38]	0.425	1.12 [0.82–1.41]	1.14 [0.83–1.40]	0.844
Prothrombin Time, median (IQR), s	13.70 [12.10–16.70]	12.70 [11.70–14.20]	<0.01	13.80 [12.10–16.90]	12.80 [11.70–14.40]	<0.01
International Normalized Ratio, median (IQR)	1.16 [1.03–1.43]	1.07 [0.99–1.19]	<0.01	1.17 [1.03–1.43]	1.07 [0.99–1.21]	<0.01
D-Dimer, median (IQR), mg/L	1.11 [0.40–3.45]	4.07 [1.43–9.61]	<0.01	1.28 [0.31–4.04]	2.63 [0.64–7.75]	0.049

Data are presented as median (interquartile range) or frequency (percentage), as appropriate. For continuous variables, normality was assessed using the Shapiro-Wilk test; normally distributed variables were compared using Student’s t-test, and non-normally distributed variables were compared using the Mann-Whitney U test. Categorical variables were compared using the chi-square test or Fisher’s exact test, as appropriate. P < 0.05 was considered statistically significant. IQR, interquartile range; AFP, alpha-fetoprotein; ALP, alkaline phosphatase; ALT, alanine aminotransferase; AST, aspartate aminotransferase; BASO%, basophil percentage; eGFR, estimated glomerular filtration rate; GGT, gamma-glutamyl transferase; HDL-C, high-density lipoprotein cholesterol; INR, international normalized ratio; LDL-C, low-density lipoprotein cholesterol; MLR, monocyte-to-lymphocyte ratio; RBP, retinol-binding protein; SSA, serum sialic acid. Additional abbreviations are defined in Table 2.

**TABLE 2:** Abbreviations.

Abbreviation	Full name
AASLD	American association for the study of liver diseases
AFU	Alpha-fucosidase
AFP	Alpha-fetoprotein
AGR	Albumin-to-globulin ratio
ALB	Albumin
ALP	Alkaline phosphatase
ALT	Alanine aminotransferase
AST	Aspartate aminotransferase
AUC	Area under the Curve
BASO%	Basophil percentage
BUN	Blood urea nitrogen
CI	Confidence interval
CK	Creatine kinase
CK-MB	Creatine kinase-MB
Cr	Creatinine
CRP	C-reactive protein
DBIL	Direct bilirubin
DCA	Decision curve analysis
DD	D-Dimer
EASL	European association for the study of the liver
eGFR	Estimated glomerular filtration rate
GGT	Gamma-glutamyl transferase
GLB	Globulin
GLOBOCAN	Global cancer observatory
HBDH	Alpha-hydroxybutyrate dehydrogenase
HCC	Hepatocellular carcinoma
HDL	High-density lipoprotein
HGB	Hemoglobin
IBIL	Indirect bilirubin
ICD-10	International classification of diseases, 10th revision
INR	International normalized ratio
IQR	Interquartile range
KNN	K-nearest neighbors
LASSO	Least absolute shrinkage and selection operator
LDH	Lactate dehydrogenase
LDL	Low-density lipoprotein
MAFLD	Metabolic dysfunction-associated fatty liver disease
MCH	Mean corpuscular hemoglobin
MCHC	Mean corpuscular hemoglobin concentration
MCV	Mean corpuscular volume
ML	Machine learning
MLR	Monocyte-to-lymphocyte ratio
MPV	Mean platelet volume
PCT	Plateletcrit
PDW	Platelet distribution width
PLT	Platelet count
PT	Prothrombin time
RBP	Retinol binding protein
RDW	Red cell distribution width
ROC	Receiver operating characteristic
SHAP	SHapley Additive exPlanations
SSA	Serum sialic acid
SVM	Support vector machine
TBIL	Total bilirubin
TC	Total cholesterol
TG	Triglycerides
TP	Total protein
UA	Uric acid
WBC	White blood cell count
XGBoost	eXtreme Gradient Boosting

### 3.2 Model construction and performance comparison

Feature selection was performed using LASSO regression, with the minimum-error criterion (lambda.min; λ = 8.49 × 10^−4^), rather than lambda.1se, selected through 5-fold cross-validation (Figure 1; Supplementary Table S3; Supplementary Figure S2). At lambda.min, 57 nonzero model terms were retained. These included serum sialic acid (SSA), basophil percentage (BASO%), monocyte-to-lymphocyte ratio (MLR), alkaline phosphatase (ALP), retinol-binding protein (RBP), the aspartate aminotransferase-to-alanine aminotransferase ratio (AST/ALT), LDH, GGT, INR, PDW, FIB, ALT, PTA, IBIL, UA, BUN/Cr, P, NEUT%, CK-MB, TBA, and the additional terms listed in Supplementary Table S3. The maximum VIF was 6.77, and no retained term exceeded the conventional VIF >10 threshold (Supplementary Table S3; Supplementary Figure S1). In the bootstrap sensitivity analysis, 49 retained terms had a selection frequency of at least 0.80; the principal predictors highlighted in the interpretation analysis, including SSA, BASO%, ALP, RBP, and AST/ALT, were consistently retained. The six classification models were then constructed using the retained terms.

**FIGURE 4:**
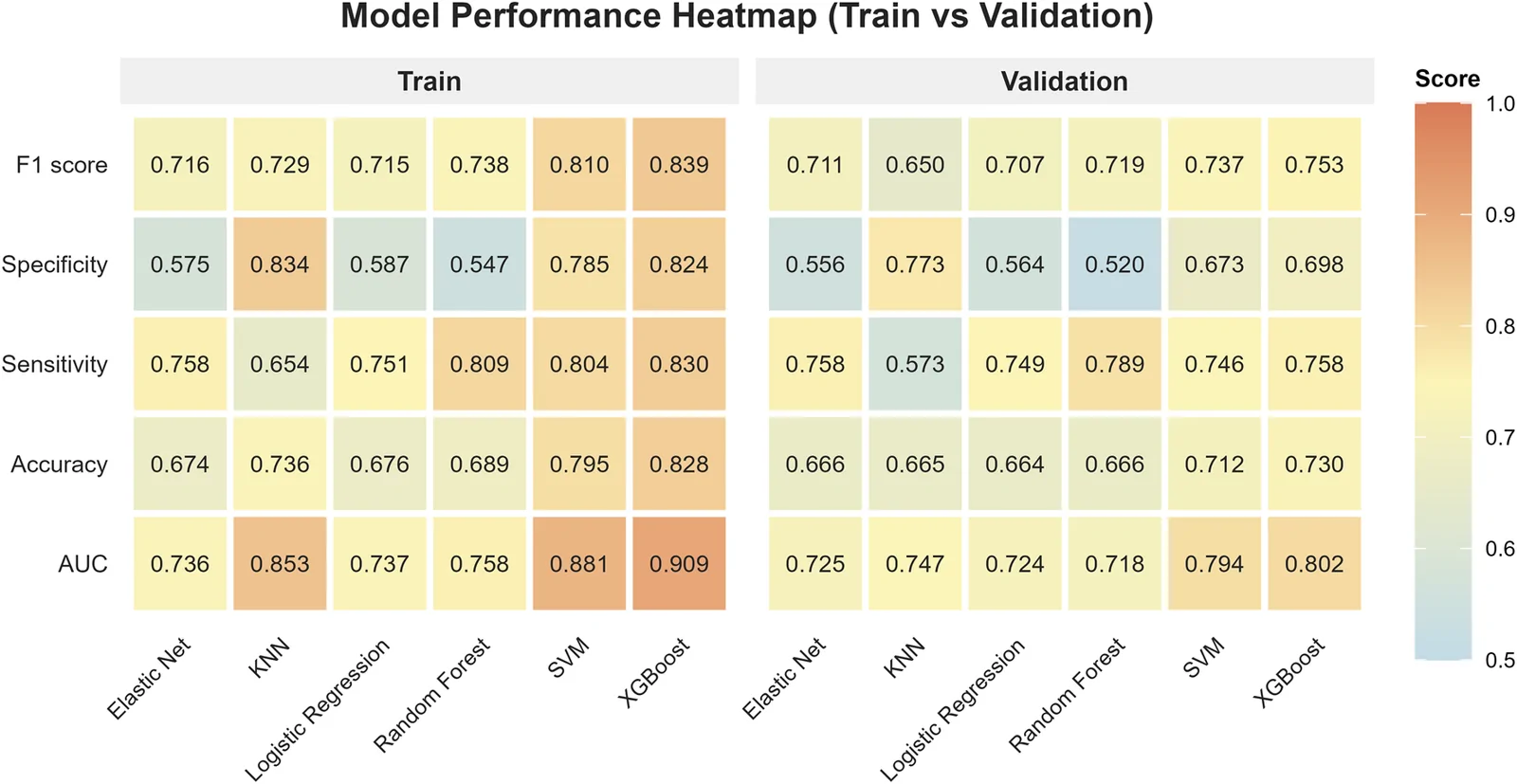
Heatmap of model performance in the training and internal validation cohorts. Rows show F1 score, specificity, sensitivity, accuracy, and the area under the receiver operating characteristic curve (AUC), and columns show the six machine-learning models within each cohort. Threshold-dependent metrics were calculated at the prespecified probability threshold of 0.5.

**FIGURE 5:**
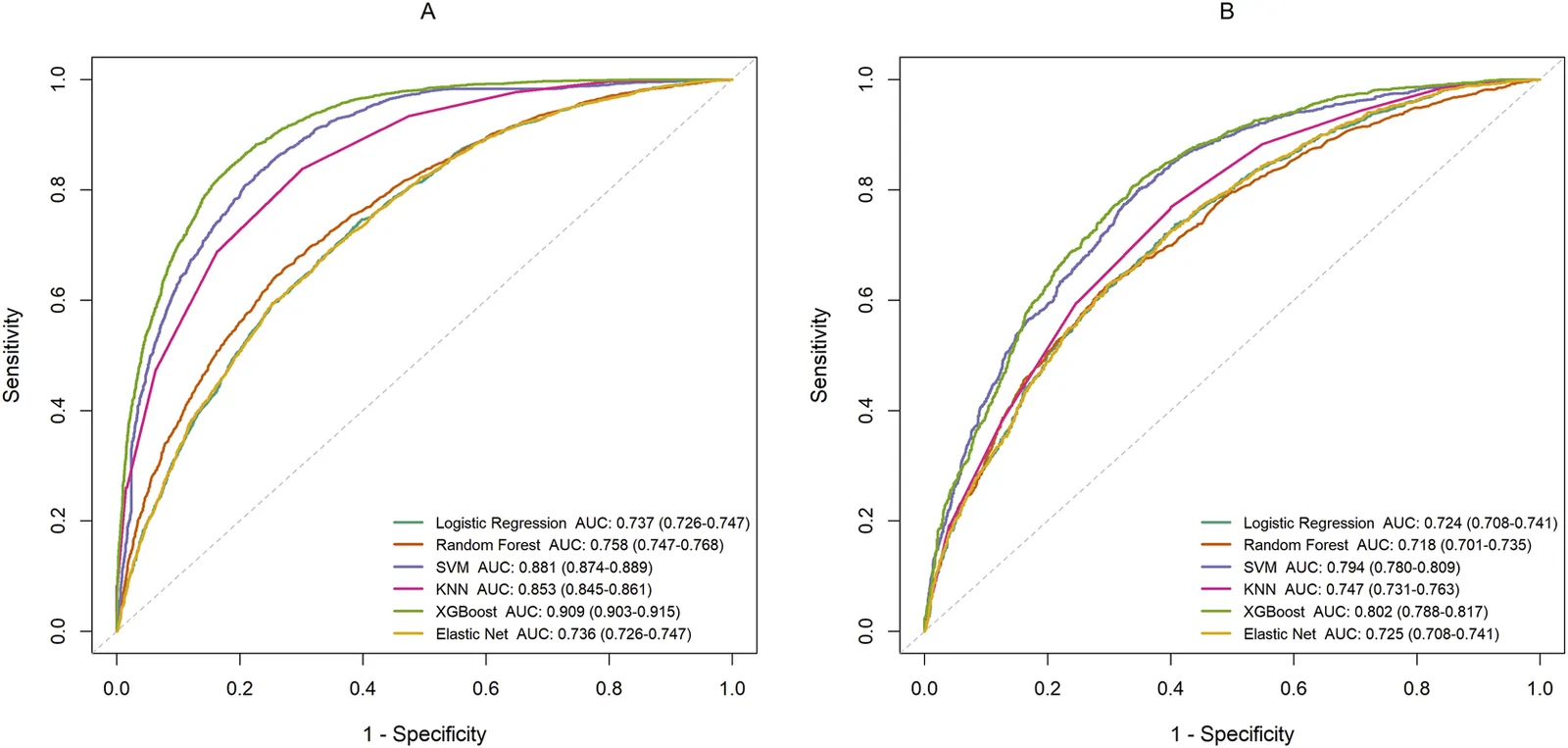
Receiver operating characteristic curves for the six machine-learning models in the training and internal validation cohorts. The x-axis represents 1 − specificity, and the y-axis represents sensitivity. The corresponding AUC values quantify model discrimination. **(A)** shows the training cohort, and **(B)** shows the internal validation cohort.

Model performance in the training and internal validation sets is summarized in Figures 4, 5. In the training set, XGBoost demonstrated the highest AUC, achieving 0.909 (95% CI, 0.903–0.915), followed by SVM and KNN. In the internal validation set, XGBoost also had the highest AUC (0.802; 95% CI, 0.788–0.817), while SVM ranked second with an AUC of 0.794. The validation-set AUCs for random forest, logistic regression, KNN, and Elastic Net were 0.718, 0.724, 0.747, and 0.725, respectively. At the prespecified probability threshold of 0.5, XGBoost achieved validation-set accuracy, sensitivity, specificity, and F1 score values of 0.730, 0.758, 0.698, and 0.753, respectively. Exploratory threshold analyses, including Youden-index and sensitivity-prioritized strategies, are presented in Supplementary Table S4; these candidate thresholds require prospective validation before clinical implementation. Pairwise DeLong testing in the validation cohort showed that XGBoost had a higher AUC than logistic regression, random forest, KNN, and Elastic Net (all P < 0.001), and a modestly higher AUC than SVM (P = 0.037; Benjamini-Hochberg-adjusted P = 0.046) (Supplementary Table S1).

### 3.3 Clinical utility analysis

**FIGURE 6:**
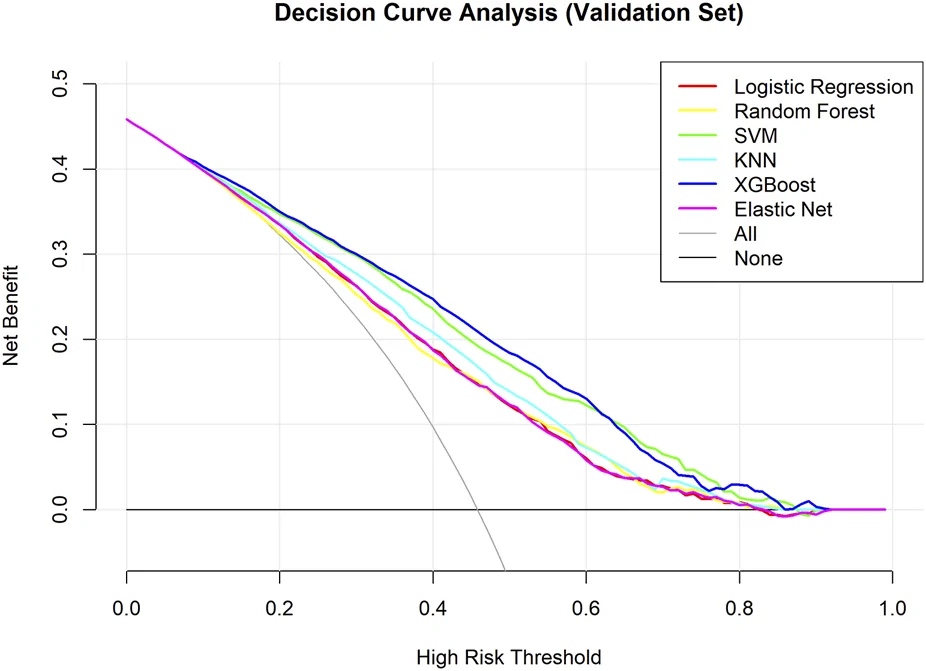
Decision curve analysis of the six machine-learning models in the internal validation cohort. The x-axis represents threshold probability, and the y-axis represents net benefit. The treat-all and treat-none strategies are shown as reference lines. A higher net benefit indicates greater potential clinical utility at the corresponding threshold.

DCA was used to evaluate the net clinical benefit of each model across risk thresholds (Figure 6). Across a wide range of threshold probabilities, XGBoost showed a higher net benefit than the other models, indicating a potentially more favorable trade-off between false-positive and false-negative classifications in this hospitalized cohort. SVM showed favorable performance in the high-risk threshold range (>0.6), whereas logistic regression, Elastic Net, and KNN yielded relatively low net benefits across most thresholds. Exploratory threshold analysis showed the expected trade-off: lowering the threshold increased sensitivity and NPV but reduced specificity and PPV. The optimal threshold should therefore be selected according to the intended clinical setting and validated prospectively.

### 3.4 Model explanation

The SHAP method and the built-in gain metric of XGBoost were used to evaluate feature importance for the optimal model. Features were ranked by mean absolute SHAP value (Figure 2). The five leading features were SSA, the AST/ALT ratio, ALP, BASO%, and MLR. The SHAP summary plot displays both the magnitude and direction of feature contributions at the individual-sample level. Higher values of SSA, the AST/ALT ratio, and ALP were mainly located on the positive side of the SHAP-value axis, indicating contributions toward a higher model-predicted probability of liver cancer. These patterns represent model-derived associations and should not be interpreted as causal biological effects. The gain metric, which quantifies the average improvement in tree-split performance attributed to a feature, ranked SSA, BASO%, MLR, ALP, and RBP as the five leading features (Figure 3); SSA ranked first by both importance measures.

Taken together, SSA, the AST/ALT ratio, ALP, BASO%, and MLR emerged as influential model predictors spanning liver function, inflammatory status, and metabolic or nutritional domains. Their importance supports their contribution to model discrimination within this hospitalized cohort but does not establish independent causal mechanisms.

## 4 Discussion

In this study, we integrated 74 multidimensional clinical and laboratory indicators from a real-world hospitalized cohort and constructed liver cancer prediction models using six machine-learning algorithms. XGBoost achieved the highest AUC in the internal validation set, and pairwise DeLong testing supported a statistically significant AUC advantage over the other evaluated models within this cohort. Decision curve analysis further suggested that XGBoost provided the greatest net clinical benefit across a range of threshold probabilities. SHAP and gain-based analyses identified SSA, the AST/ALT ratio, ALP, BASO%, and MLR as influential predictors. These results support the potential value of routine clinical and laboratory indicators for liver cancer risk-prediction model development.

The XGBoost model showed stronger internal validation performance than logistic regression, random forest, Elastic Net, and KNN, and its AUC was also slightly but significantly higher than that of SVM in the paired DeLong test (P = 0.037). To define the novelty of this work more explicitly, we compared the present study with representative liver cancer prediction tools and AI models in terms of population, data source, endpoint, and performance context (Supplementary Table S6). GALAD is an established biomarker score based on sex, age, AFP, AFP-L3, and des-gamma-carboxy prothrombin (Johnson et al., 2014; Best et al., 2020), whereas HCC-Scope and related neural-network approaches have focused on specific etiologic populations such as nonalcoholic steatohepatitis-related hepatocellular carcinoma (Okanoue et al., 2023). Other recent AI studies have relied on digital pathology, CT, MRI, or treatment-response settings and have often addressed recurrence, prognosis, therapeutic response, or imaging-defined phenotypes rather than risk prediction from routine inpatient data (Wang et al., 2026; Zhang et al., 2024; Huang et al., 2025; Liu et al., 2025). In contrast, the present study was developed from a large Hainan hospitalized real-world cohort, used routine electronic medical record and laboratory indicators, incorporated hospitalized non-liver cancer controls with a mapped disease spectrum, and identified SSA and other routine composite inflammatory or liver-function indicators as influential predictors. However, because direct head-to-head comparisons with established clinical biomarkers or scores such as AFP and GALAD were not available in this retrospective dataset, these results do not establish superiority over existing screening strategies, and the incremental clinical value of the model remains to be tested in external, outpatient, first-visit, and prospective cohorts.

Feature-importance analysis identified SSA, the AST/ALT ratio, ALP, BASO%, and MLR as key model predictors. SSA ranked first in both the SHAP and gain-based analyses, suggesting that it provided substantial information for discrimination in this cohort. Supplementary Table S5 provides descriptive context for the top 10 XGBoost features, including SSA, in the liver-cancer and non-liver-cancer groups. SSA was higher in the liver-cancer group in both the training and validation cohorts. This pattern may reflect inflammatory, metabolic, hepatobiliary, or disease-severity information captured by the model, but it does not establish SSA as a replacement for AFP or as a causal biomarker. Future studies should directly compare the model with AFP, GALAD, and other established tools and assess whether SSA provides incremental discrimination in AFP-negative or early-stage liver cancer.

From a clinical perspective, the model developed from large-scale inpatient data suggests that routinely collected variables may help construct low-cost liver cancer risk-prediction tools. Because these variables are commonly available, the modeling framework does not require specialized equipment or expensive assays. However, the present cohort consisted exclusively of hospitalized patients, and the case proportion was much higher than expected in community-based screening. Therefore, the model should not yet be interpreted as a validated primary-care or population-screening tool. Before broader deployment, validation is required in outpatient first-visit patients, high-risk follow-up populations, community-based cohorts, and external centers, together with prospective assessment of clinically meaningful thresholds.

This study has several limitations. First, the data were derived from a single tertiary hospital and exclusively from hospitalized patients, which may introduce selection and spectrum bias; performance may therefore be overestimated for community-based screening. Second, the near-balanced liver-cancer proportion differs from real-world prevalence and may affect calibration, PPV, NPV, and generalizability. Third, no independent external validation cohort was available. Fourth, BCLC stage information was not available, precluding stage-stratified and early-stage-specific evaluation. Fifth, a formal head-to-head comparison with AFP, GALAD, or guideline-recommended surveillance strategies could not be performed because AFP was incomplete and AFP-L3 and des-gamma-carboxy prothrombin were unavailable. Sixth, the disease-spectrum summary was based on diagnosis records rather than unique-patient counts. Seventh, although VIF analysis did not indicate severe multicollinearity, SHAP values remain model-based contributions rather than independent causal effects. Future studies should prospectively validate the model in outpatient and first-visit populations, community-based high-risk groups, external multicenter cohorts, and BCLC- or early-stage-stratified subgroups, with direct comparison against established screening tools and assessment of calibration in representative target populations.

## 5 Conclusion

In this study, an XGBoost-based prediction model for liver cancer was constructed using routine clinical indicators from a real-world hospitalized population. The model showed satisfactory discrimination and net clinical benefit in internal validation, supporting the feasibility of developing liver cancer risk-prediction models from large-scale routine laboratory data. Key predictors, including serum sialic acid (SSA), the aspartate aminotransferase-to-alanine aminotransferase ratio (AST/ALT), and alkaline phosphatase (ALP), contributed importantly to model performance. Further validation in outpatient and first-visit patients, stage-stratified evaluation, external validation, and comparison with established screening tools are required before the model can be applied to broader early-risk identification or population-based screening.

## Data Availability

The raw data supporting the conclusions of this article will be made available by the authors, without undue reservation.
