# Supplementary material for: Machine learning models for predicting liver cancer: a real-world cohort study in China
Source: Front Cell Dev Biol. 2026 Sep 9;14:1896989. doi: 10.3389/fcell.2026.1896989 (PMC13597679; doi:10.3389/fcell.2026.1896989)
Supplement: Supplementary file 1 [file Table1.docx]

**Supplementary Table S1. Pairwise DeLong comparison between XGBoost and other models in the internal validation cohort**

Paired DeLong tests were performed using model-predicted probabilities. XGBoost AUC was 0.802 in the finalized manuscript. After verification against the final ROC-analysis output and the corresponding AUC summary, the KNN comparator AUC and displayed AUC difference were corrected. P values were adjusted using the Benjamini-Hochberg method; displayed AUC differences are calculated from the rounded AUC values.

| **Comparator** | **XGBoost AUC** | **Comparator AUC** | **AUC difference** | **P value** | **BH-adjusted P value** |
| --- | --- | --- | --- | --- | --- |
| Logistic regression | 0.802 | 0.724 | 0.078 | <0.001 | <0.001 |
| Random forest | 0.802 | 0.718 | 0.084 | <0.001 | <0.001 |
| SVM | 0.802 | 0.794 | 0.008 | 0.037 | 0.046 |
| KNN | 0.802 | 0.747 | 0.055 | <0.001 | <0.001 |
| Elastic Net | 0.802 | 0.725 | 0.077 | <0.001 | <0.001 |

Abbreviations: AUC, area under the receiver operating characteristic curve; BH, Benjamini-Hochberg; SVM, support vector machine; KNN, k-nearest neighbors.
