# Supplementary material for: Machine learning models for predicting liver cancer: a real-world cohort study in China
Source: Front Cell Dev Biol. 2026 Sep 9;14:1896989. doi: 10.3389/fcell.2026.1896989 (PMC13597679; doi:10.3389/fcell.2026.1896989)
Supplement: Supplementary file 2 [file Table2.docx]

**Supplementary Table S2. Diagnosis-record spectrum of non-liver-cancer records before application of the final exclusion criteria**

This table summarizes 6,336 non-liver-cancer diagnosis records from the source cohort before application of the final exclusion criteria. It describes the initial diagnosis-record spectrum and is not the denominator of the final modeling cohort. Counts are diagnosis-record counts and should not be interpreted as unique-patient counts. The final model cohort included 4,760 non-liver-cancer controls (3,296 in training and 1,464 in internal validation).

| **Disease category** | **n (records)** | **%** |
| --- | --- | --- |
| Cirrhosis / chronic liver disease | 2167 | 34.2 |
| Viral hepatitis with cirrhosis | 1672 | 26.4 |
| Unspecified diagnosis | 524 | 8.3 |
| Viral hepatitis | 417 | 6.6 |
| Endocrine, metabolic, or nutritional diseases | 250 | 3.9 |
| Symptoms, signs, follow-up, or history codes | 233 | 3.7 |
| Other malignant tumors or metastatic tumors | 212 | 3.3 |
| Other digestive system diseases | 208 | 3.3 |
| Biliary or pancreatic diseases | 193 | 3.0 |
| Other conditions | 184 | 2.9 |
| Liver failure / decompensated liver disease | 110 | 1.7 |
| Cardiovascular or cerebrovascular diseases | 101 | 1.6 |
| Respiratory diseases | 39 | 0.6 |
| Genitourinary or kidney diseases | 26 | 0.4 |
