# Supplementary material for: Machine learning models for predicting liver cancer: a real-world cohort study in China
Source: Front Cell Dev Biol. 2026 Sep 9;14:1896989. doi: 10.3389/fcell.2026.1896989 (PMC13597679; doi:10.3389/fcell.2026.1896989)
Supplement: Supplementary file 3 [file Table3.docx]

**Supplementary Table S3. LASSO feature selection, multicollinearity diagnosis, and selection stability**

The minimum-error criterion (lambda.min; λ = 8.49 × 10⁻⁴) retained 57 nonzero model terms. The maximum VIF was 6.77, no retained term had VIF >10, and 49 retained terms had bootstrap selection frequency ≥0.80.

| **Feature** | **Model term** | **Coefficient** | **VIF** | **Selection frequency** |
| --- | --- | --- | --- | --- |
| Upper_Gastrointestinal_Bleeding | Upper_Gastrointestinal_Bleeding1 | -0.7579 | 1.04 | 0.98 |
| Liver_Failure | Liver_Failure1 | -0.3678 | 1.38 | 1.00 |
| PDW | PDW | -0.3085 | 2.91 | 1.00 |
| NEUT% | NEUT. | 0.2832 | 3.05 | 1.00 |
| Hepatic_Encephalopathy | Hepatic_Encephalopathy1 | 0.2630 | 1.15 | 0.94 |
| INR | INR | -0.2517 | 1.70 | 1.00 |
| RBP | RBP | -0.2502 | 3.09 | 1.00 |
| SSA | SSA | 0.2434 | 4.43 | 1.00 |
| ALP | ALP | 0.2249 | 1.70 | 1.00 |
| BASO% | BASO. | 0.2249 | 1.30 | 1.00 |
| TBA | TBA | -0.2224 | 2.01 | 1.00 |
| GLU | GLU | -0.2188 | 1.59 | 1.00 |
| IBIL | IBIL | -0.2007 | 3.76 | 1.00 |
| AST/ALT | AST.ALT | 0.1632 | 1.21 | 1.00 |
| AFU | AFU | 0.1604 | 1.97 | 1.00 |
| Cr | Cr | -0.1578 | 4.74 | 0.99 |
| Cl | Cl | -0.1500 | 5.14 | 1.00 |
| P_LCR | P_LCR | 0.1484 | 2.95 | 1.00 |
| UA | UA | 0.1449 | 1.96 | 1.00 |
| Ca | Ca | -0.1302 | 1.78 | 1.00 |
| MCH | MCH | 0.1025 | 2.22 | 1.00 |
| P | P | -0.1001 | 1.81 | 1.00 |
| PTA | PTA | 0.0886 | 3.04 | 0.97 |
| Na | Na | 0.0881 | 4.34 | 0.91 |
| MONO% | MONO. | 0.0846 | 2.37 | 0.95 |
| Cys_C | Cys_C | -0.0771 | 3.89 | 0.89 |
| D_3_HB | D_3_HB | -0.0666 | 1.33 | 0.99 |
| eGFR | eGFR | -0.0658 | 2.67 | 0.94 |
| CPK | CPK | 0.0650 | 1.53 | 0.94 |
| K | K | 0.0620 | 1.57 | 0.97 |
| BUN/Cr | BUN.Cr | 0.0614 | 3.89 | 0.92 |
| TT | TT | -0.0602 | 1.51 | 0.97 |
| FIB | FIB | 0.0590 | 2.93 | 0.95 |
| LDL/HDL | LDL.HDL | 0.0561 | 3.11 | 0.92 |
| Liver_Cirrhosis | Liver_Cirrhosis1 | 0.0557 | 1.07 | 0.92 |
| PCT | PCT | -0.0545 | 2.34 | 0.89 |
| ALT | ALT | -0.0520 | 1.84 | 0.92 |
| TG | TG | -0.0476 | 1.85 | 0.97 |
| RDW | RDW | 0.0455 | 2.24 | 0.91 |
| LDH | LDH | 0.0448 | 2.24 | 0.77 |
| ASO | ASO | -0.0418 | 1.11 | 0.97 |
| GSP | GSP | 0.0368 | 3.55 | 0.73 |
| CK_MB | CK_MB | 0.0350 | 1.73 | 0.87 |
| LDL_C | LDL_C | 0.0258 | 6.77 | 0.75 |
| TB/DB | TB.DB | -0.0246 | 1.62 | 0.85 |
| TCO2 | TCO2 | 0.0240 | 2.36 | 0.80 |
| Urea | Urea | 0.0240 | 6.60 | 0.63 |
| HDL_C | HDL_C | 0.0232 | 2.64 | 0.83 |
| Mg | Mg | 0.0187 | 1.34 | 0.87 |
| GGT | GGT | -0.0176 | 1.88 | 0.77 |
| Ascites | Ascites1 | 0.0175 | 1.11 | 0.82 |
| ApoB | ApoB | -0.0148 | 6.59 | 0.66 |
| MCHC | MCHC | 0.0143 | 1.97 | 0.75 |
| MLR | MLR | 0.0131 | 2.56 | 0.80 |
| SII | SII | -0.0092 | 2.47 | 0.71 |
| Lp(a) | Lp.a. | -0.0042 | 1.32 | 0.83 |
| HCY | HCY | 0.0038 | 1.56 | 0.82 |

Abbreviations: LASSO, least absolute shrinkage and selection operator; VIF, variance inflation factor. Software-safe model-term names are retained where applicable.
