# Supplementary material for: Machine learning models for predicting liver cancer: a real-world cohort study in China
Source: Front Cell Dev Biol. 2026 Sep 9;14:1896989. doi: 10.3389/fcell.2026.1896989 (PMC13597679; doi:10.3389/fcell.2026.1896989)
Supplement: Supplementary file 4 [file Table4.docx]

**Supplementary Table S4. Threshold-dependent performance of the XGBoost model in the internal validation cohort**

The prespecified 0.5-threshold metrics are harmonized to the finalized main manuscript. The Youden-index and sensitivity-prioritized thresholds are exploratory candidate decision thresholds and require prospective validation.

| **Threshold approach** | **Threshold** | **Accuracy** | **Sensitivity** | **Specificity** | **PPV** | **NPV** | **F1 score** |
| --- | --- | --- | --- | --- | --- | --- | --- |
| Prespecified 0.50 threshold | 0.500 | 0.730 | 0.758 | 0.698 | 0.705 | 0.753 | 0.753 |
| Youden-index threshold | 0.475 | 0.730 | 0.744 | 0.718 | 0.691 | 0.768 | 0.716 |
| Sensitivity-prioritized threshold | 0.378 | 0.716 | 0.850 | 0.602 | 0.644 | 0.826 | 0.733 |

Abbreviations: PPV, positive predictive value; NPV, negative predictive value; F1, F1 score.
