# Supplementary material for: Machine learning models for predicting liver cancer: a real-world cohort study in China
Source: Front Cell Dev Biol. 2026 Sep 9;14:1896989. doi: 10.3389/fcell.2026.1896989 (PMC13597679; doi:10.3389/fcell.2026.1896989)
Supplement: Supplementary file 5 [file Table5.docx]

**Supplementary Table S5. Baseline distributions of the top 10 XGBoost features**

Features are ordered according to the gain-based XGBoost importance ranking. Values are median [interquartile range]. The table uses the finalized patient-level training and internal validation cohort definitions; overlapping variables are shown on the same original clinical scale as Table 1 of the main manuscript.

| **Cohort** | **Rank** | **Feature** | **Feature label** | **Non-liver cancer** | **Liver cancer** |
| --- | --- | --- | --- | --- | --- |
| Training cohort | 1 | SSA | Serum sialic acid | 0.69 [0.53-0.90] | 0.78 [0.60-0.99] |
| Training cohort | 2 | BASO% | Basophil percentage (%) | 0.40 [0.20-0.70] | 0.50 [0.30-0.70] |
| Training cohort | 3 | MLR | Monocyte-to-lymphocyte ratio | 0.39 [0.26-0.65] | 0.48 [0.33-0.76] |
| Training cohort | 4 | ALP | Alkaline phosphatase (U/L) | 89.80 [66.95-131.25] | 113.60 [78.90-183.12] |
| Training cohort | 5 | RBP | Retinol-binding protein (mg/L) | 14.31 [8.32-22.88] | 16.38 [10.25-23.79] |
| Training cohort | 6 | AST/ALT | AST/ALT ratio | 1.29 [0.92-1.80] | 1.44 [1.05-2.13] |
| Training cohort | 7 | LDH | Lactate dehydrogenase (U/L) | 219.40 [181.55-274.35] | 243.95 [199.10-327.65] |
| Training cohort | 8 | GGT | Gamma-glutamyl transferase (U/L) | 50.60 [25.90-111.90] | 90.10 [44.70-177.50] |
| Training cohort | 9 | INR | International normalized ratio | 1.16 [1.03-1.43] | 1.07 [0.99-1.19] |
| Training cohort | 10 | PDW | Platelet distribution width (fL) | 13.50 [11.60-15.80] | 12.50 [11.00-14.30] |
| Validation cohort | 1 | SSA | Serum sialic acid | 0.70 [0.51-0.89] | 0.77 [0.59-0.99] |
| Validation cohort | 2 | BASO% | Basophil percentage (%) | 0.40 [0.20-0.60] | 0.50 [0.30-0.70] |
| Validation cohort | 3 | MLR | Monocyte-to-lymphocyte ratio | 0.40 [0.27-0.67] | 0.49 [0.33-0.77] |
| Validation cohort | 4 | ALP | Alkaline phosphatase (U/L) | 89.30 [67.00-129.20] | 114.65 [79.62-180.93] |
| Validation cohort | 5 | RBP | Retinol-binding protein (mg/L) | 14.05 [8.08-22.57] | 15.78 [9.65-23.61] |
| Validation cohort | 6 | AST/ALT | AST/ALT ratio | 1.29 [0.94-1.78] | 1.45 [1.04-2.17] |
| Validation cohort | 7 | LDH | Lactate dehydrogenase (U/L) | 220.40 [183.60-285.10] | 244.75 [198.25-318.62] |
| Validation cohort | 8 | GGT | Gamma-glutamyl transferase (U/L) | 53.50 [26.63-115.87] | 88.50 [44.05-163.00] |
| Validation cohort | 9 | INR | International normalized ratio | 1.17 [1.03-1.43] | 1.07 [0.99-1.21] |
| Validation cohort | 10 | PDW | Platelet distribution width (fL) | 13.60 [11.70-16.10] | 12.50 [11.10-14.40] |

Abbreviations: SSA, serum sialic acid; BASO%, basophil percentage; MLR, monocyte-to-lymphocyte ratio; ALP, alkaline phosphatase; RBP, retinol-binding protein; AST/ALT, aspartate aminotransferase-to-alanine aminotransferase ratio; LDH, lactate dehydrogenase; GGT, gamma-glutamyl transferase; INR, international normalized ratio; PDW, platelet distribution width.
