# Supplementary material for: Machine learning models for predicting liver cancer: a real-world cohort study in China
Source: Front Cell Dev Biol. 2026 Sep 9;14:1896989. doi: 10.3389/fcell.2026.1896989 (PMC13597679; doi:10.3389/fcell.2026.1896989)
Supplement: Supplementary file 6 [file Table6.docx]

**Supplementary Table S6. Comparison with representative liver cancer prediction tools and AI models**

This table summarizes representative prior tools or AI studies cited in the manuscript. The comparison clarifies population, data source, endpoint, and contribution; it does not imply direct superiority across non-identical study designs.

| **Study or tool** | **Population or setting** | **Main variables or data source** | **Endpoint or reported performance** | **Comparison with the present study** |
| --- | --- | --- | --- | --- |
| GALAD model (Refs. 13, 14) | Patients at risk for hepatocellular carcinoma, including chronic liver disease and nonalcoholic steatohepatitis cohorts in prior validation studies. | Sex, age, AFP, AFP-L3, and des-gamma-carboxy prothrombin. | Early hepatocellular carcinoma detection; prior studies reported strong diagnostic performance in surveillance-oriented cohorts. | GALAD is an established biomarker score, whereas the present study used routine electronic medical record and laboratory variables. AFP-L3 and des-gamma-carboxy prothrombin were unavailable in our dataset, so formal GALAD calculation was not possible. |
| Okanoue et al. / HCC-Scope neural-network model (Ref. 19) | Patients with nonalcoholic steatohepatitis or metabolic dysfunction-associated fatty liver disease backgrounds. | A neural-network model using clinical and serologic factors, including age, sex, liver enzymes, lipid variables, platelet count, diabetes-related information, and AIM-related markers. | Nonalcoholic steatohepatitis-related hepatocellular carcinoma detection; the original manuscript cited sensitivity 85.3% and specificity 85.1%. | This prior model was disease-etiology specific. The present study was derived from a large Hainan hospitalized cohort with a broader non-liver cancer control spectrum and used routinely available multidimensional laboratory indicators. |
| Digital pathology or postoperative recurrence models (Refs. 16, 17) | Patients with hepatocellular carcinoma after surgery or with available pathological material. | Pathomics, histopathological images, and selected clinical variables. | Postoperative recurrence or prognosis prediction rather than first identification of liver cancer. | These models address prognostic tasks and require pathology or surgical specimens. The present model was built from pre-existing routine inpatient clinical and laboratory data. |
| Treatment-response machine-learning model (Ref. 28) | Patients with unresectable hepatocellular carcinoma receiving targeted and immune therapy. | Clinical and CT-derived variables before treatment. | Pretreatment prediction of response to targeted and immune therapy; the cited study reported validation AUC around 0.80. | The endpoint was therapeutic response in confirmed hepatocellular carcinoma, whereas the present study focused on liver cancer prediction using routine clinical and laboratory indicators. |
| Gadoxetic acid-enhanced MRI interpretable machine-learning model (Ref. 29) | Patients with hepatocellular carcinoma undergoing gadoxetic acid-enhanced MRI. | MRI features interpreted with machine-learning and SHAP approaches. | Identification of cholangiocyte-phenotype hepatocellular carcinoma and related phenotype/prognostic tasks. | This approach depends on specialized imaging, whereas the present study used inexpensive routine variables that are easier to extract repeatedly from hospital information systems. |
| Present study | 9,284 hospitalized patients from Hainan General Hospital: 6,426 in the training cohort and 2,858 in the internal validation cohort; hospitalized non-liver-cancer controls had a mapped diagnosis-record spectrum. | Seventy-four candidate demographic, electronic medical record, and laboratory variables; 57 nonzero LASSO model terms at lambda.min, of which 49 had bootstrap selection frequency ≥0.80; six machine-learning algorithms. | Internal-validation liver cancer prediction; XGBoost AUC 0.802 (95% CI, 0.788-0.817), paired DeLong comparisons, DCA, threshold analysis, and SHAP interpretation. | The contribution is a routine-data model developed and interpreted in a large Hainan hospitalized cohort, with mapped control-disease spectrum and explicit internal-validation analyses. The study does not establish superiority over GALAD or population-screening strategies. |

Abbreviations: AFP, alpha-fetoprotein; AFP-L3, Lens culinaris agglutinin-reactive alpha-fetoprotein; AUC, area under the receiver operating characteristic curve; DCA, decision curve analysis; GALAD, gender-age-AFP-L3-AFP-DCP score; SHAP, SHapley Additive exPlanations; SSA, serum sialic acid; XGBoost, extreme gradient boosting.
